# *GhAGL16* (*AGAMOUS*-*LIKE16*) Negatively Regulates Tolerance to Water Deficit in Transgenic *Arabidopsis* and Cotton

**DOI:** 10.3390/plants13020282

**Published:** 2024-01-18

**Authors:** Jianfeng Lei, Yangzi You, Peihong Dai, Li Yu, Yue Li, Chao Liu, Xiaodong Liu

**Affiliations:** 1 College of Agronomy, Xinjiang Agricultural University, Nongda East Road, Urumqi 830052, China; kyleijianfeng@163.com; 2 College of Life Sciences, Xinjiang Agricultural University, Nongda East Road, Urumqi 830052, China; youyangzi@126.com (Y.Y.); peihong816@163.com (P.D.); yulixjnu@163.com (L.Y.); liyue6905@126.com (Y.L.); liuch_86@126.com (C.L.)

**Keywords:** MADS-box transcription factor, *GhAGL16*, water stress, stomata, ABA signaling

## Abstract

Cotton is one of the most economically important crops in the world, and drought is a key abiotic factor that can significantly reduce cotton yield. MADS-box transcription factors play essential roles in various aspects of plant growth and development as well as responses to biotic and abiotic stress. However, the use of MADS-box transcription factors to regulate water stress responses has not been fully explored in cotton. Here, we showed that GhAGL16 acts as a negative regulator of water deficit in cotton, at least in part by regulating ABA signaling. *GhAGL16*-overexpressing (*GhAGL16*-OE) transgenic *Arabidopsis* had lower survival rates and relative water contents (RWCs) under water stress. Isolated leaves of *GhAGL16*-OE *Arabidopsis* had increased water loss rates, likely attributable to their increased stomatal density. *GhAGL16*-OE *Arabidopsis* also showed reduced primary root lengths in response to mannitol treatment and decreased sensitivity of seed germination to ABA treatment. By contrast, silencing *GhAGL16* in cotton enhanced tolerance to water deficit by increasing proline (Pro) content, increasing superoxide dismutase (SOD) and peroxidase (POD) activities, and reducing malondialdehyde (MDA) and hydrogen peroxide (H_2_O_2_) contents under water stress. Subcellular localization and transcriptional activation assays confirmed that GhAGL16 is a nuclear protein that lacks transcriptional self-activation activity. The expression of ABA biosynthesis-related genes (*GhNCED3/7/14*), a catabolism-related gene (*GhCYP707A*), and a gene related to the ABA signaling pathway (*GhABF4*) was altered in *GhAGL16*-silenced plants. Taken together, our data demonstrate that GhAGL16 plays an important role in cotton resistance to water stress.

## 1. Introduction

Cotton is an economically important crop in China, and its growth and development are subject to a range of environmental stresses. Among these stressors, drought stands out as the primary abiotic factor that significantly constrains both growth and yield [1]. Drought triggers a multifaceted signaling network in plants, which culminates in the deployment of adaptive responses [2]; improving drought resistance is therefore a pivotal strategy for ensuring the stability of cotton yield. One common approach involves the breeding of a novel, drought-resistant germplasm through the overexpression of genes that positively regulate drought resistance. These include the transcriptional regulatory genes *AREB*, *NAC*, *DREB*, *NF*-*Y*, and *WRKY*; genes involved in post-translational protein modifications such as *CDPK*, *SnRK2*, and *MAPK*; the metabolic regulatory genes *LOS*, *NCED*, and *PLC*; and genes associated with osmotic stress protection like *LEA*, *P5CS*, *TPS*, and *betA* [3]. In addition to these positive regulators, a number of plant genes also act as negative regulators of drought resistance, such as *MdSE* [4], *AtCOST1* [5], *OsWRKY114* [6], and *OsWRKY5* [7].

The complex regulatory mechanisms that have evolved to respond to drought stress include specific transcriptional activators and repressors that modulate the expression of downstream genes. Among these are the MADS-box proteins, members of a widespread family of eukaryotic transcription factors that regulate various aspects of plant growth, development, and biotic and abiotic stress responses. MADS-box proteins are divided into two categories, type I and type II, on the basis of their conserved structural domains [8]. Most MADS-box proteins with known functions in plants belong to type II, also known as the MIKC type because they consist of MADS (M), I, K, and C-terminal domains [9]. Type II MADS-box transcription factors have important roles in the regulation of root growth, floral meristem development, and the vegetative-to-reproductive transition [10,11,12,13], and several studies demonstrated their key regulatory roles in response to various abiotic stresses. For example, tomato *SlMBP22* overexpression lines exhibited improved drought tolerance attributed to changes in auxin and gibberellin signaling [14]. OsMADS57 directly binds to the *OsWRKY94* gene promoter and activates its transcription in response to cold stress, and overexpression of *OsMADS57* maintains rice tiller growth under low-temperature stress [15]. *OsMADS26* was found to negatively regulate disease and drought resistance in rice [16], whereas *OsMADS23* positively regulated salt and drought tolerance [17]. Identification and characterization of wheat MADS-box transcription factors revealed that many of them show changes in expression under abiotic and biotic stress [18]. Although a substantial portion of MADS-box proteins have been functionally characterized in various crops, research on their role in cotton stress responses remains limited.

The AGL16 (AGAMOUS-LIKE16) transcription factor is a member of the type II MADS-box family, and it is mainly expressed in leaf guard cells and trichomes of *Arabidopsis* [19]. Previous studies reported that *AGL16* transcripts are targeted by *miR824*, and both mutation of *AGL16* and overexpression of *miR824* were linked to reduced stomatal numbers [20,21]. *AGL16* is also involved in the unequal distribution of stomata [22], and AGL16 interacts with MYB44 to regulate seed dormancy and germination in *Arabidopsis* [23]. AGL16 plays a negative regulatory role in the response to drought stress in *Arabidopsis* through transcriptional inhibition of *AAO3* (ABA biosynthesis pathway-related gene) and *SDD1* (stomatal density- and distribution-related gene), activation of *CYP707A3* (ABA catabolic pathway-related gene) to regulate leaf stomatal density and ABA content [24]. It also functions as a negative regulator of the salt-stress response by downregulating salt-responsive genes [25]. Importantly, a frameshift mutation in *AGL16* produced no substantial changes in growth and development of the mutant compared with the wild type (WT), suggesting that this gene is a promising candidate for breeding of drought- and salt-tolerant crops.

To date, the potential function of AGL16 in cotton under drought stress has not been characterized. Previously, we found that *GhAGL16* is predominantly expressed in cotton leaves (leaves > roots > stems), consistent with the expression pattern of *AGL16* in *Arabidopsis* [19]. *GhAGL16* expression was also responsive to water deficit, high salt, and ABA treatments [26]. Therefore, we speculated that *GhAGL16* might participate in the response of cotton to water stress. To assess this possibility, we investigated the function of *GhAGL16* in *Arabidopsis* and cotton under water stress and obtained preliminary evidence that *GhAGL16* negatively regulates water deficit tolerance. These results provide a foundation for further studies of the molecular mechanism(s) by which *GhAGL16* regulates cotton drought resistance.

## 2. Materials and Methods

### 2.1. Plant Materials and Growth Conditions

Seeds of the upland cotton variety Xinluzao 22 and *N. benthamiana* were sterilized, sown in nutrient soil, and cultured in a growth room at 28 °C with a 16 h/8 h light/dark photoperiod. All *Arabidopsis* materials used in this study were the Columbia ecotype (Col-0). Seeds of Col-0, *AtAGL16*-OE, and *GhAGL16*-OE were sterilized and vernalized on 1/2 Murashige and Skoog (MS) medium at 4 °C for 3 days. After the seedlings had rooted and the two cotyledons had expanded, they were transplanted into nutrient soil and cultured at 22 °C with a 16 h/8 h light/dark photoperiod.

### 2.2. Sequence Analysis

In our previous work, we cloned the full-length, 720 bp open reading frame (ORF) of *GhAGL16* from Xinluzao 22 (this germplasm was expanded and preserved in our laboratory). Here, we aligned the amino acid sequences of GhAGL16 and AtAGL16 and analyzed their homology and conserved structural domains using DNAMAN version 8.0.

### 2.3. Subcellular Localization and Transcriptional Activation Analysis

To determine the subcellular localization of GhAGL16, we cloned the full-length coding sequence of *GhAGL16* into the pCAMBIA1304 vector to generate the 35S::*GhAGL16*-GFP-p1304 construct. 35S::*GhAGL16*-GFP-p1304 (or the pCAMBIA1304 empty vector) and Histone 2B, fused with red fluorescent mCherry protein (H2B-mCherry) [27], were transformed into *A. tumefaciens* GV3101 for transient expression in 3-week-old *N. benthamiana* leaves. The fluorescence was observed by laser confocal microscopy (Zeiss, Oberkochen, Germany) 2 days after injection.

To assess the transcriptional activation activity of GhAGL16, the full-length coding sequence of *GhAGL16* was cloned into pGBKT7 to generate the pGBKT7-GhAGL16 construct containing the GAL4 DNA-binding domain. The pGBKT7-AtDREB construct [28] was used as a positive control. The two recombinant plasmids and the negative control pGBKT7-BD empty vector were transferred into yeast AH109, which was then cultured on SD/-Trp medium for 3 days. Successfully transformed yeast strains were diluted to different concentrations and transferred to SD/-Trp and SD/-Trp-His-Ade media for 3–5 days for observation of colony growth.

### 2.4. Transformation of Arabidopsis Plants

The full-length coding sequences of *AtAGL16* and *GhAGL16* were recombined into the 35SN-pCAMBIA1301 vector to generate the 35S::*AtAGL16*-p1301 and 35S::*GhAGL16*-p1301 constructs. These two recombinant plasmids were transferred into *A. tumefaciens* GV3101 for transformation of *Arabidopsis* by the floral dip method [29]. Transgenic seedlings were screened for resistance to hygromycin (30 µg/mL) on 1/2 MS agar medium, and transgenic plants were confirmed by GUS histochemistry. To further confirm the stable transgenic plants, relative expression levels of *AtAGL16* and *GhAGL16* genes were measured by RT–qPCR. *AtActin2* was used as the internal reference. T_3_-generation plants with the highest transgene expression were used for subsequent experiments.

### 2.5. Water Stress Treatment and Determination of Physiological and Biochemical Indicators

T_3_-generation *AtAGL16*-OE, *GhAGL16*-OE, and WT *Arabidopsis* plants were selected for water stress treatment. Before water stress treatment, each pot was filled with 80 g of nutrient soil, and *Arabidopsis* seedlings of similar size were transplanted into the pots. Water (100 mL) was replenished once during the growth process, and other environmental parameters remained consistent. 3-week-old *Arabidopsis* plants were then exposed to natural water stress by withholding water for the indicated times. Phenotypic differences before and after treatment were photographed, and the survival rate was calculated. Leaf RWC was measured before and after water stress treatment by a previously described method [30]. The rate of water loss from fresh leaves of nonstressed plants was measured by weighing. Epidermal cells were obtained from the abaxial leaf surface of WT and *GhAGL16*-OE plants by a previously described method [31], and the number of stomata was observed and imaged under a fluorescence inverted microscope (Leica, Wetzlar, Germany). The number of stomata in 10 random fields of view was counted for each leaf to calculate stomatal density. The contents of proline (Pro), hydrogen peroxide (H_2_O_2_), and malondialdehyde (MDA) and the activities of superoxide dismutase (SOD) and peroxidase (POD) in leaves were measured according to the instructions of commercial kits (Solarbio, Beijing, China).

### 2.6. Determination of Seed Germination Rate and Root Length

WT and T_3_-generation *GhAGL16*-OE *Arabidopsis* seeds were sown on MS medium supplemented with different ABA concentrations (0.5 and 1 μM); three sets of replicates were set up, and the number of germinated seeds was counted each day. In addition, seedlings of similar size were selected and transferred to MS medium with different mannitol concentrations (200 or 300 mM); root length was photographed and measured after 7 days of vertical growth.

### 2.7. VIGS (Virus-Induced Gene Silencing)

VIGS was performed as described previously [28]. A silencing fragment (293 bp) of *GhAGL16* was recombined with the linearized TRV:RNA2 vector to generate the TRV:*GhAGL16* construct. *GhCLA1*, whose silencing is easily confirmed by an albino phenotype, was selected as the target for construction of TRV:*GhCLA1* as a visual marker for monitoring of silencing efficiency [32]. TRV:*GhAGL16*, TRV:*GhCLA1*, and the TRV:RNA1 and TRV:RNA2 empty vectors were transfected into *A. tumefaciens* GV3101 for use in transformation of cotton seedlings with two fully unfolded cotyledons. After inoculation with TRV:*GhCLA1*, leaves showed an albino phenotype, and young leaves from the corresponding plants were sampled for RT–qPCR to check the efficiency of target gene silencing.

### 2.8. RNA Isolation and RT–qPCR (Real-Time Quantitative PCR)

Tissue samples of cotton and *Arabidopsis* were rapidly frozen in liquid nitrogen. Total RNA was extracted using the TRIzol reagent (Transgen, Beijing, China) according to the manufacturer’s instructions. First-strand cDNA was synthesized from RNA using the TransScript Reverse Transcriptase kit (Transgen) according to the manufacturer’s instructions. RT–qPCR was performed on an ABI 7500 system (Applied Biosystems, Life Technologies, Carlsbad, CA, USA) using TransStart Green qPCR SuperMix (Transgen) following the manufacturer’s instructions. The relative fold changes in transcript levels were calculated using the 2^−ΔΔCt^ method. *GhUBQ7* was used as the internal reference gene for cotton, and *AtActin2* was used for *Arabidopsis*. All primers are shown in Table S1.

### 2.9. Analysis of GUS Activity

The transgenic plants were stained as described previously [33] with GUS staining solution prepared as described in Lei et al. [34].

### 2.10. Statistical Analysis

Values are the mean ± standard deviation (SD) of three biological replicates. Statistical analysis was performed using Student’s *t*-test.

## 3. Results

### 3.1. Identification and Sequence Characteristics of GhAGL16

**Figure 1:**
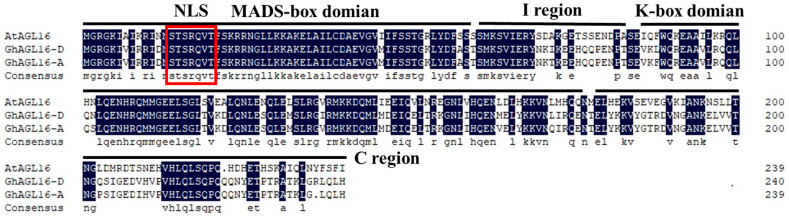
Multiple sequence alignment of GhAGL16-A, GhAGL16-D, and AtAGL16. The black background indicates identical amino acids. The conserved MADS-box domain, I region, K-box domain, and C-terminal region are underlined. The red box indicates the nuclear localization signal (NLS).

The MADS-box transcription factor AtAGL16 acts as a negative regulator of drought resistance in *Arabidopsis*. To explore the potential roles of its cotton homologs, we isolated and cloned *GhAGL16-A* and *GhAGL16-D* from the A and D subgenomes of Xinluzao 22. The GhAGL16-A protein consisted of 239 amino acids, and the GhAGL16-D protein consisted of 240 amino acids. Homology assessments revealed that GhAGL16-A and GhAGL16-D shared 96.65% amino acid similarity with one another and 64.02% and 63.75% similarity, respectively, with *Arabidopsis* AGL16. Alignment of GhAGL16-A/D with AtAGL16 showed that both GhAGL16 proteins contain a nuclear localization signal (NLS), an N-terminal conserved MADS-box domain, and a central, relatively conserved K-box domain typical of type II MADS-box proteins (Figure 1). These findings suggest that GhAGL16 and AtAGL16 may share similar biological functions. Given their high amino acid identity, we treated GhAGL16-A and GhAGL16-D as a single protein hereafter. We chose the *GhAGL16-A* sequence for further research.

### 3.2. GhAGL16 Is a Nuclear Protein with No Transcriptional Self-Activating Activity

**Figure 2:**
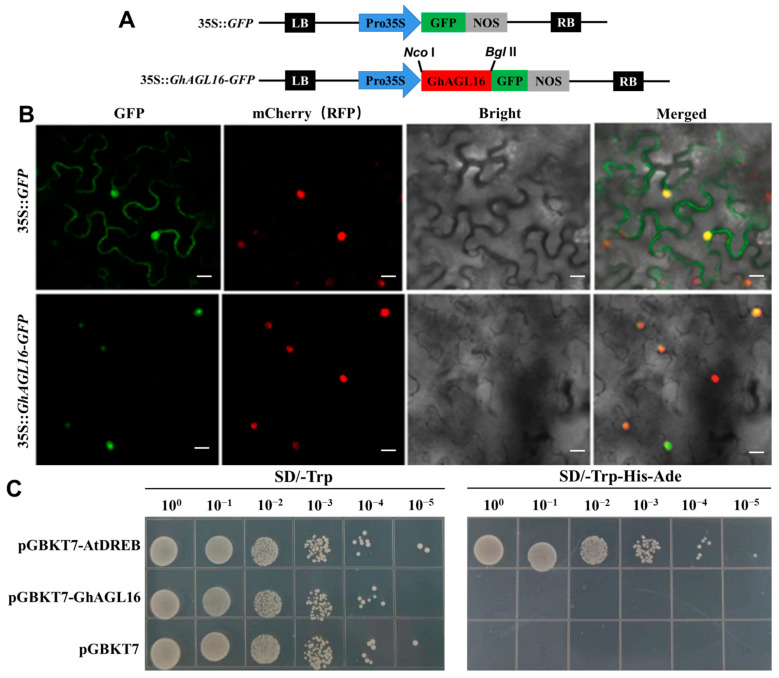
Subcellular localization and transcriptional activation assays of GhAGL16. (**A**) The GFP-fusion vector (35S::*GhAGL16*-*GFP*) and control construct (35S::*GFP*) used for transient transformation of *N. benthamiana*. (**B**) Subcellular localization of GhAGL16. 35S::*GhAGL16*-*GFP* and mCherry constructs were co-transformed into *N. benthamiana* leaves via *A. tumefaciens*. 35S::*GFP* was used as the control. Scale bars, 20 µm. (**C**) Transcriptional activation activity analysis of GhAGL16. The pGBKT7-AtDREB construct and the empty vector pGBKT7 were used as the positive and negative control, respectively.

To confirm this subcellular localization, we generated a 35S::*GhAGL16*-*GFP* construct (Figure 2A) and co-transformed it into *N. benthamiana* with mCherry as a nuclear marker; 35S::*GFP* was used as the control. Observation of GFP fluorescence with a confocal laser scanning microscope confirmed that GFP alone localized to both the nucleus and cell membrane, whereas *GhAGL16-GFP* and mCherry both localized only to the nuclei (Figure 2B). These results indicate that GhAGL16 is a nuclear-localized protein.

To examine the transcriptional activation activity of GhAGL16, we first assessed its transcriptional self-activating activity in yeast cells. Neither yeast expressing pGBKT7-GhAGL16 nor those expressing the negative control (pGBKT7) grew on SD/-Trp-His-Ade medium, whereas those expressing the positive control pGBKT7-AtDREB grew on this medium (Figure 2C). These results indicate that GhAGL16 does not have transcriptional self-activating activity in yeast cells.

### 3.3. Screening and Identification of Transgenic Plants

**Figure 3:**
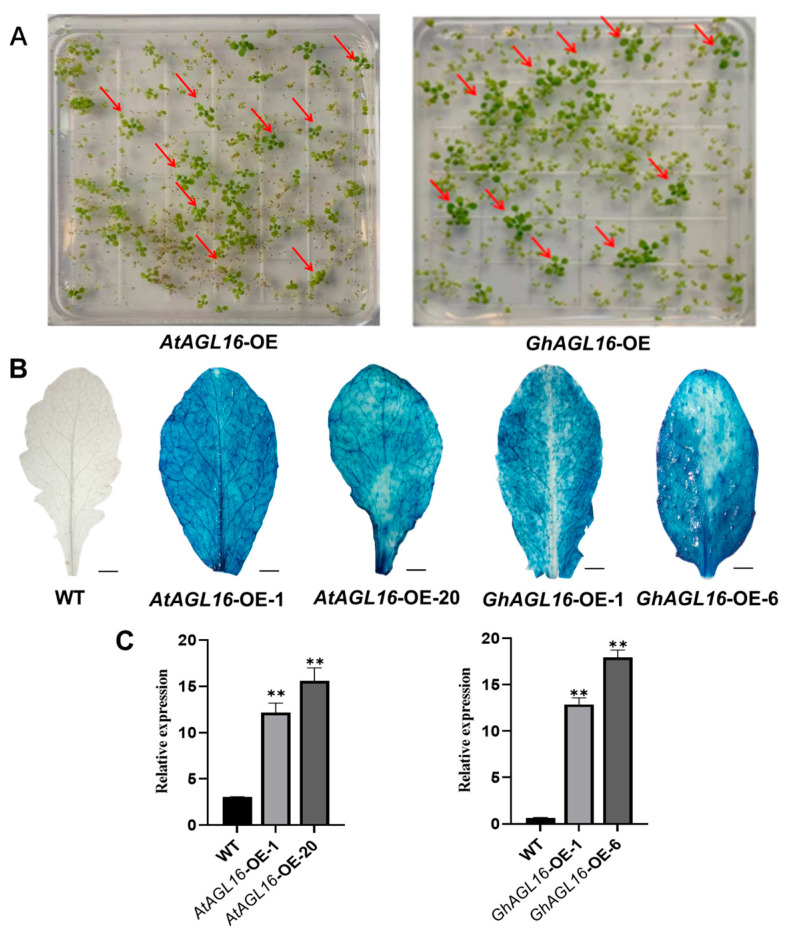
Screening and identification of *AtAGL16*-OE and *GhAGL16*-OE transgenic *Arabidopsis.* (**A**) Hygromycin screening of transgenic plants. Red arrows indicate positive transgenic plants. (**B**) GUS histochemical staining of *Arabidopsis* leaves. Scale bars, 3 cm (**C**) Expression of *AtAGL16* and *GhAGL16* in the corresponding transgenic *Arabidopsis* lines. *AtActin2* was used as the reference gene. Values are the mean ± SD (*n* = 3 replicates). ** *p* < 0.01 (Student’s *t*-test).

*AtAGL16*-OE and *GhAGL16*-OE transgenic *Arabidopsis* seedlings were screened on 1/2 MS medium containing 30 µg/mL hygromycin. Seedlings that rooted normally and produced green cotyledons were preliminarily identified as positive transgenic plants (Figure 3A). GUS staining of the positive transgenic plants showed that the *AtAGL16*-OE and *GhAGL16*-OE lines displayed different degrees of blue leaf staining (Figure 3B), indicating that the *GUS* gene was stably expressed in these lines. *AtAGL16* and *GhAGL16* expression was examined by RT–qPCR. *AtAGL16* expression was higher (approximately 10- to 15-fold) in *AtAGL16*-OE-1 and *AtAGL16*-OE-20 compared with the WT. Similarly, *GhAGL16* expression was higher (approximately 10- to 20-fold) in *GhAGL16*-OE-1 and *GhAGL16*-OE-6 compared with the WT (Figure 3C). On the basis of these results, seeds of positive transgenic plants were collected and propagated to the T_3_ generation for subsequent study.

### 3.4. Overexpression of GhAGL16 Reduced Tolerance to Water Deficit in Arabidopsis

**Figure 4:**
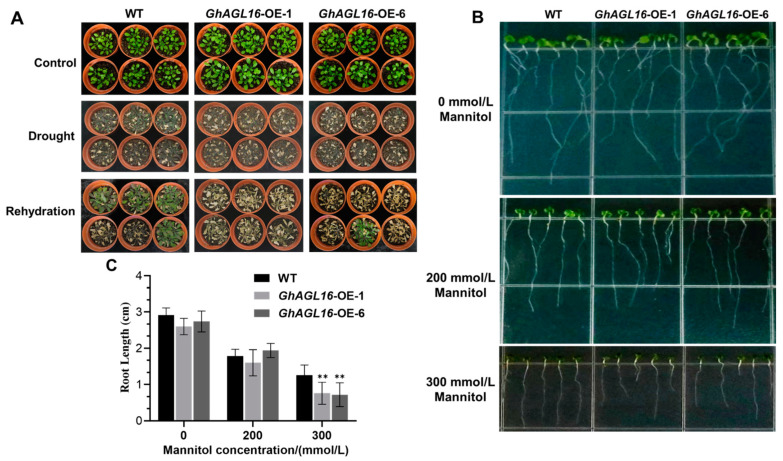
Overexpression of *GhAGL16* decreases tolerance to water deficit in *Arabidopsis*. (**A**) Phenotype of *GhAGL16*-OE and WT plants after drought and rehydration treatments. 3-week-old soil-grown seedlings of WT and *GhAGL16*-OE plants were subjected to water stress for 15 days and rehydration for 3 days. Values are the mean ± SD (*n* = 3 replicates, 24 seedlings per replicate). (**B**,**C**) Primary root elongation. Seeds of WT and *GhAGL16*-OE plants were germinated on MS medium, transferred to MS medium with or without mannitol (200 or 300 mM), and grown vertically for another 7 days. Photographs were obtained (**B**), and primary root length was measured (**C**). Values are the mean ± SD (*n* = 3 replicates, 5 seedlings per replicate). ** *p* < 0.01 (Student’s *t*-test).

We next performed a water deficit tolerance assay on soil-grown *Arabidopsis* plants to evaluate the function of *GhAGL16* in the water stress response. Under normal growth conditions, there were no visible differences among WT, *GhAGL16*-OE-1, and *GhAGL16*-OE-6 lines. When 3-week-old, soil-grown plants were subjected to water stress treatment for 15 days, the *GhAGL16*-OE-1 and *GhAGL16*-OE-6 lines had lower survival rates (0% and 16.7%, respectively) than the WT (66.7%). After 3 days of rehydration, the two transgenic lines showed irreversible death, whereas most of the WT plants slowly resumed normal growth (Figure 4A). These results indicate that overexpression of *GhAGL16* reduces the tolerance to water deficit in *Arabidopsis*. The drought-resistance phenotypes of the *AtAGL16*-OE (OE-1 and OE-20) lines were basically consistent with those of the *GhAGL16*-OE lines (Supplementary Figure S1). Under water stress, MDA and H_2_O_2_ contents were significantly higher in *GhAGL16*-OE and *AtAGL16*-OE plants than in WT plants, and proline contents and SOD and POD activities were significantly lower. By contrast, there were no clear differences in these parameters under normal growth conditions (Supplementary Figure S2). Together, these results suggest that *GhAGL16* and *AtAGL16* have a similar, negative effect on the water-stress response in *Arabidopsis*.

To confirm the role of GhAGL16 in stress response, we performed primary root elongation assays. Seeds of WT and *GhAGL16*-OE plants were germinated on MS medium for 3 days, transferred to MS medium with or without mannitol (200 or 300 mM), and grown vertically for another 7 days. Under normal growth conditions (MS) and 200 mM mannitol treatment, there was no difference in primary root length between WT and *GhAGL16*-OE plants. Primary root length of all plants was reduced in response to 200 and 300 mM mannitol. However, *GhAGL16*-OE plants showed a greater reduction in root length than WT plants in response to 300 mM mannitol (Figure 4B,C). These results provide evidence that GhAGL16 negatively effects primary root elongation under abiotic stress.

### 3.5. Overexpression of GhAGL16 Increased Stomatal Density and Water Loss in Arabidopsis

**Figure 5:**
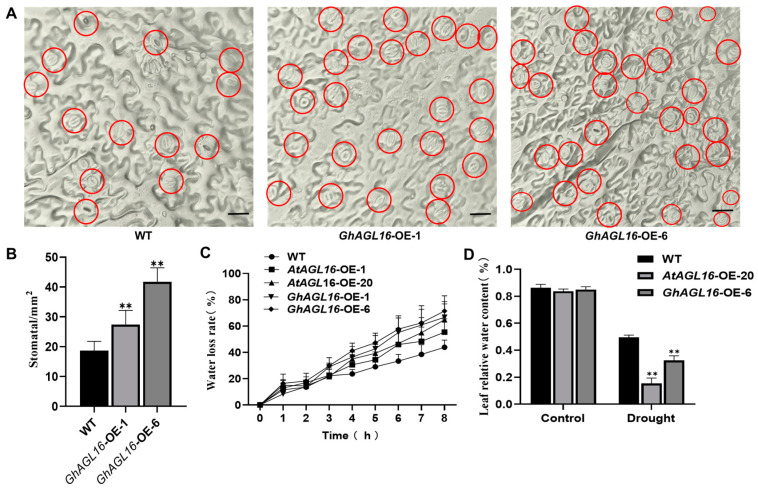
Overexpression of *GhAGL16* increased stomatal density in *Arabidopsis*. (**A**) Stomatal density of rosette leaves from the same position in WT and *GhAGL16*-OE lines; red circles highlight the stomata. Scale bars, 10 µm. (**B**) Stomatal density of WT and *GhAGL16*-OE lines. Values are means ± SD (*n* = 3 plants per genotype, 10 images analyzed per plant). (**C**) Water loss rate (%) of detached fifth rosette leaves from 3-week-old, soil-grown WT and *GhAGL16*-OE plants. Leaves were allowed to dry at 22 °C and 60% relative humidity. Values are mean ± SD (*n* = 3 replicates). (**D**) Leaf relative water content (RWC). 4-week-old soil-grown seedlings were subjected to water stress by withholding water for 14 days. Values are mean ± SD (*n* = 3 replicates, 8 plants per genotype). ** *p* < 0.01 (Student’s *t*-test).

Stomata play an important role in regulating water loss during water stress, and about 90% of water in plants is lost through stomatal transpiration [35]. To further investigate the mechanism by which *GhAGL16* affects *Arabidopsis* drought resistance, we investigated stomatal density, a major determinant of drought tolerance. *GhAGL16*-OE plants had a higher stomatal density than the WT under normal conditions and after 15 days of water stress treatment (Figure 5A,B). To estimate transpiration under water stress, we measured the water loss rate of detached rosette leaves. *GhAGL16*-OE leaves showed a higher rate of water loss than WT leaves at multiple time points (Figure 5C). The *GhAGL16*-OE plants also had a lower RWC than WT plants after water stress treatment (Figure 5D). Thus, overexpression of *GhAGL16* in *Arabidopsis* appears to increase stomatal density and water loss, results that correlate well with the water stress-response phenotypes of the different lines and indicate that GhAGL16 negatively regulates tolerance to water deficit in *Arabidopsis*.

### 3.6. Overexpression of GhAGL16 Decreased the Sensitivity of Arabidopsis Germination to ABA

**Figure 6:**
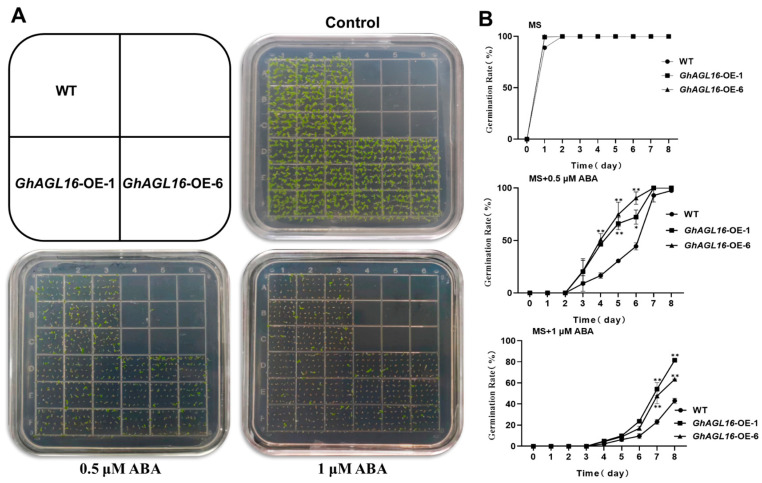
Overexpression of *GhAGL16* decreased the sensitivity of *Arabidopsis* seed germination to ABA. (**A**) Seed germination of WT and *GhAGL16*-OE plants. Seeds were germinated horizontally on Murashige and Skoog (MS) medium with 0, 0.5, or 1 µM ABA for 4 days. (**B**) Seed germination rate was measured at the indicated time points. Values are mean ± SD (*n* = 3 replicates, 180 seeds per replicate). * *p* < 0.05, ** *p* < 0.01 (Student’s *t*-test).

In previous work, we found that expression of *GhAGL16* was induced by ABA, suggesting that *GhAGL16* may be involved in ABA-related signaling pathways. We therefore performed seed germination assays to investigate whether *GhAGL16* was involved in ABA-mediated inhibition of germination. WT and *GhAGL16*-OE seeds were germinated on MS medium with 0, 0.5, or 1 μM ABA. In the absence of ABA, seed germination rate did not differ among genotypes. By contrast, germination rate was higher for *GhAGL16*-OE seeds than for WT seeds under 0.5 and 1.0 μM ABA (Figure 6A,B). Thus, overexpression of *GhAGL16* reduces the sensitivity of seeds to germination inhibition by ABA.

### 3.7. Silencing of GhAGL16 in Cotton Improved Water Deficit Resistance

**Figure 7:**
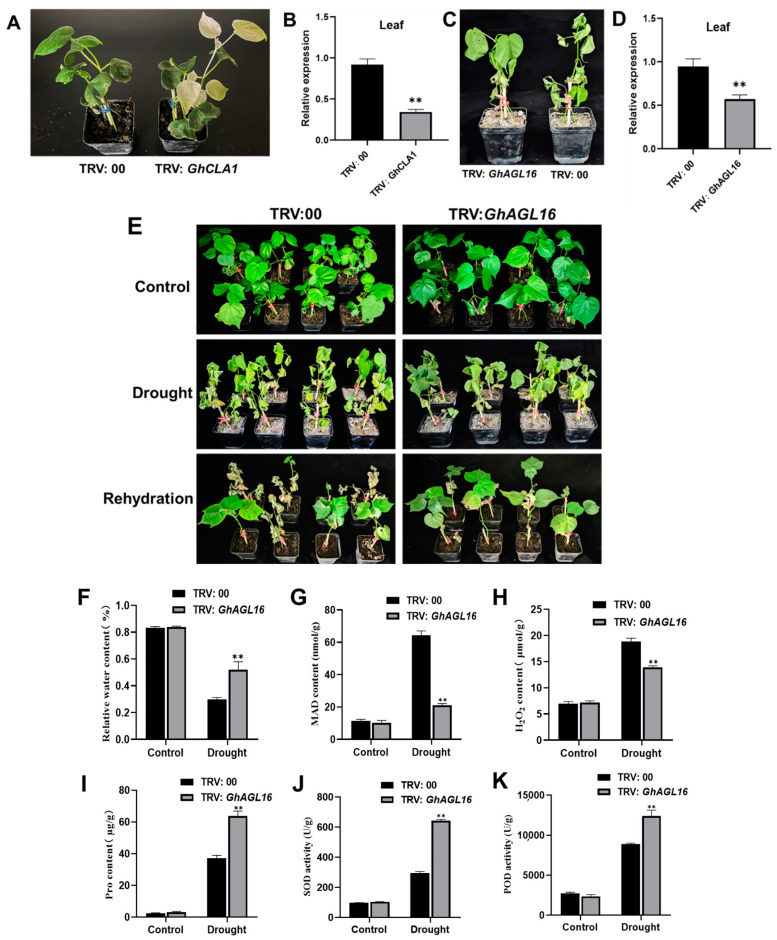
Virus-induced silencing of *GhAGL16* enhances tolerance to water deficit in cotton. (**A**) Phenotypes of *GhCLA1* silencing in cotton. (**B**) Expression of *GhCLA1* in TRV:00 and TRV:*GhCLA1* plants. (**C**) Phenotypes of *GhAGL16* silencing in cotton under water stress. (**D**) Expression of *GhAGL16* in TRV:00 and TRV:*GhAGL16* plants. (**E**) Phenotypes of TRV:00 and TRV:*GhAGL16* plants under drought stress and after rehydration. (**F**–**K**) Drought-related biochemical indexes: RWC (**F**), MDA content (**G**), H_2_O_2_ content (**H**), Pro content (**I**), SOD activity (**J**), and POD activity (**K**). Values are mean ± SD (*n* = 3 replicates). ** *p* < 0.01, (Student’s *t*-test).

To further explore the role of *GhAGL16* in cotton drought resistance, we generated *GhAGL16*-silenced plants (*GhAGL16-A* and *GhAGL16-D*) in the Xinluzao 22 background using a VIGS technique. 15 days after VIGS vector inoculation, leaves of positive control TRV:*GhCLA1* plants showed an albino phenotype, whereas the negative control TRV:00 showed no changes (Figure 7A). RT–qPCR revealed significant downregulation of *GhCLA1* in leaves (Figure 7B), confirming that the TRV-VIGS system worked as expected. We also confirmed successful silencing of *GhAGL16* in cotton (Figure 7C,D). Under normal growth conditions, there were no visible differences between TRV:00 and TRV:*GhAGL16* plants. By contrast, TRV:*GhAGL16* plants exhibited higher survival than TRV:00 plants after 10 days of water stress. After 4 days of rehydration, TRV:00 plants showed irreversible death, whereas most TRV:*GhAGL16* plants slowly resumed normal growth. The survival rate of WT plants was 22.2%, whereas that of TRV:*GhAGL16* plants was 56.3% (Figure 7E). We also measured a number of physiological indexes under normal and water-stressed conditions. As shown in Figure 7F–K, there were no differences in any of these physiological parameters between TRV:00 and TRV:*GhAGL16* plants under normal conditions. After water stress, RWC, Pro content, and SOD and POD activities were significantly higher in TRV:*GhAGL16* plants than in TRV:00 plants, and MDA and H_2_O_2_ contents were significantly lower. These results demonstrate that knockdown of *GhAGL16* enhances cotton tolerance to water deficit.

### 3.8. GhAGL16 May Negatively Regulate ABA Signaling

**Figure 8:**
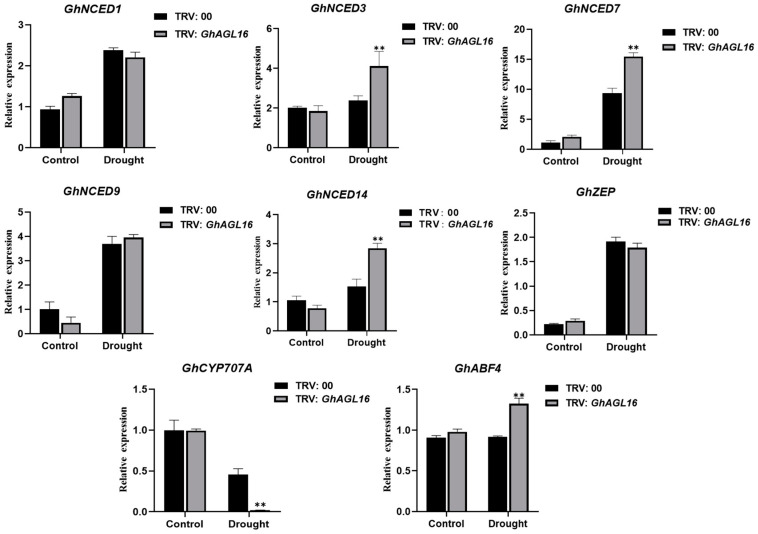
Expression of ABA-related genes in TRV:00 and TRV:*GhAGL16* plants. Values are mean ± SD (*n* = 3 replicates). ** *p* < 0.01 (Student’s *t*-test).

Plant transcription factors participate in abiotic stress signaling and metabolic pathways by activating or repressing the transcription of stress-response-related genes. We investigated changes in the expression of several genes associated with ABA metabolism and signaling pathways to explore the mechanism of enhanced water deficit tolerance in *GhAGL16*-silenced plants. After water stress treatment, three ABA biosynthesis-related genes (*GhNCED3*/*7*/*14*) and one ABA signaling pathway gene (*GhABF4*) were significantly upregulated in *GhAGL16*-silenced plants compared with TRV:00 plants, whereas an ABA catabolism-related gene (*GhCYP707A*) was significantly downregulated (Figure 8). These results suggest that *GhAGL16* may negatively regulate the resistance of cotton to water stress by modulating ABA signaling.

## 4. Discussion

The MADS-box protein AtAGL6 plays a pivotal regulatory role in leaf stomatal density, stomatal movement, ABA-mediated seed germination, and responses to salt and osmotic stress in *Arabidopsis* [24,25]. In this study, we cloned the homologous cotton MADS-box gene *GhAGL16* from leaves of Xinluzao 22. The amino acid sequence of GhAGL16 includes a highly conserved MADS-box domain, a moderately conserved I-domain, a relatively conserved K-domain, and a highly variable C-terminal domain (Figure 1). These characteristic features imply that GhAGL16 is a prototypical type II MADS-box transcription factor. Expression of *AtAGL16* decreased in response to NaCl and mannitol stress but increased in response to ABA treatment, implying that AtAGL16 participates in multiple facets of the abiotic stress response. Previous investigations showed that *GhAGL16* is expressed predominantly in cotton leaves, with relatively high expression in roots, and its expression also responds to water stress, salt, and ABA treatments [26]. These findings suggest that GhAGL16 may also function as a stress-responsive transcription factor that contributes to abiotic stress adaptation in cotton. A number of studies have shown that AtAGL16 not only participates in the drought response of *Arabidopsis* but also is closely linked to the salt-tolerance response [24,25].

The MADS-box gene family encodes transcription factors that share a highly conserved DNA-binding domain. These family members regulate diverse plant developmental processes, from root formation to fruit ripening [10,11,12,13]. A total of 207 MADS-box genes have been identified in the genome of upland cotton [36]. Nonetheless, most previous studies have focused on MADS-box genes that function in cotton floral organ development, such as *GhMADS1* [37], *GhMADS3* [38], and *GhMADS13* [39]. The specific role(s) of cotton MADS-box genes in the water-stress response remain to be clarified. Unlike other MADS-box transcription factors such as JcMADS40 [11], ZMM7-L [40], and CsMADS3 [41], GhAGL16 does not show transcriptional self-activation in yeast cells (Figure 2). This implies that GhAGL16 may need to interact with other proteins in yeast to trigger transcriptional activation.

Genotype limitations and protracted transformation cycles are the two primary technical limitations on cotton genetic transformation, constraining the development of transgenic and gene-edited plants. By contrast, transformation of the model plant *Arabidopsis* benefits from its short growth cycle, well-established genetic transformation technology, and abundant seed production. These attributes enabled us to rapidly assess the function of *GhAGL16* upon heterologous overexpression in *Arabidopsis*. Using antibiotic-based selection, GUS histochemical staining, and gene expression analysis, we produced two transgenic *Arabidopsis* lines with *GhAGL16* overexpression (*GhAGL16*-OE) (Figure 3). Compared with the WT, *GhAGL16*-OE transgenic *Arabidopsis* exhibited lower survival rates and RWC under water stress. The transgenic plants showed more rapid water loss from isolated leaves, resulting in a lower capacity to resist water deficit. This outcome was probably attributable to the increased stomatal density of the transgenic *Arabidopsis* (Figure 5A,B). Previous studies have showed that *Arabidopsis* AGL16 directly suppresses *SDD1* expression by binding to the CArG-motif (CC(A/T)GG) in the *SDD1* promoter [24]. Transgenic plants overexpressing *AtAGL16* showed a significant increase in stomatal density, consistent with the phenotype of an *Atagl16* mutant created by CRISPR-Cas9 gene-editing technology [42]. In addition, evidence suggests that overexpression of *ZmSDD1* leads to a reduction in stomatal density in maize, thereby increasing drought tolerance [43]. However, we found no changes in the *AtSDD1* transcript level in *GhAGL16*-OE transgenic *Arabidopsis*. This result implies that the effect of GhAGL16 on stomatal density may be mediated through alternative pathways. Osmotic stress functions as the primary signal associated with drought stress [44]. Here, mannitol treatment caused a reduction in the root length of *GhAGL16*-OE plants (Figure 4B,C), indicating that *GhAGL16* overexpression decreased the drought tolerance of *Arabidopsis* at the seedling stage. According to previous studies, GhAGL16 may regulate the drought response through an ABA-dependent pathway. However, contrary to expectations, germination of *GhAGL16*-OE *Arabidopsis* seeds was less sensitive to the inhibitory influence of ABA, and *GhAGL16*-OE seeds exhibited a higher germination rate under ABA treatment (Figure 6). By contrast, the expression of ABA biosynthesis-related genes increased significantly under water stress treatment in *GhAGL16*-silenced plants (Figure 8). This inconsistency may be explained by the fact that GhAGL16 transcription factors play different roles in ABA-mediated seed germination and ABA synthesis.

Previous reports suggested that stress-responsive MADS-box genes could be introduced into future crop breeding programs to improve the stress resistance of genetically modified and gene-edited crops [14,16,45] For example, tomato plants overexpressing *SlMBP11* show increased tolerance to salt stress [46], and *SlMBP8*-knockdown plants have stronger tolerance to drought and salt stress [45]. In this study, survival rate and tolerance to water deficit were significantly higher in TRV:*GhAGL16*-silenced plants than in TRV:00 plants under water stress (Figure 7E). Many studies have shown that plants typically exhibit various characteristic physiological and biochemical changes under abiotic stress [6,28]. After water stress, RWC, Pro content, and SOD and POD activities were significantly higher in TRV:*GhAGL16*-silenced plants than in TRV:00 plants, and MDA and H_2_O_2_ contents were significantly lower (Figure 7F–K). These results strongly suggest that *GhAGL16* is a negative regulatory gene for drought resistance, and knockdown of *GhAGL16* expression can enhance cotton tolerance to water deficit. We also investigated changes in the expression of marker genes related to ABA metabolism and signaling pathways in TRV:*GhAGL16*-silenced plants. After water stress, the expression of ABA biosynthesis-related genes (*GhNCED3*, *GhNCED7*, and *GhNCED14*) and an ABA signaling pathway-related gene (*GhABF4*) was significantly higher in TRV:*GhAGL16*-silenced plants than in TRV:00 plants, whereas expression of the ABA catabolism-related gene *GhCYP707A* was significantly lower. These results indicate that the expression of stress-responsive genes may increase water deficit tolerance in TRV:*GhAGL16*-silenced plants.

In this study, we used reverse genetics to investigate the biological functions and potential mechanisms by which GhAGL16 influences water deficit tolerance in *Arabidopsis* and cotton. The findings reveal that GhAGL16 acts as a suppressor of drought resistance—a discovery that posits *GhAGL16* as a promising candidate for the engineering of a novel drought-resistant cotton germplasm using gene-editing technology. This study also provides a theoretical basis for further characterization of the molecular mechanism(s) by which GhAGL16 influences water deficit tolerance.

## Data Availability

The data presented in this study are available in the article or the Supplementary Materials.

## Supplementary Materials

The following supporting information can be downloaded at: https://www.mdpi.com/article/10.3390/plants13020282/s1, Figure S1: Phenotypes of *AtAGL16*-OE and WT plants before and after drought stress and after rehydration; Figure S2: MDA, H_2_O_2_, and Pro contents as well as SOD and POD activities were determined in *GhAGL16*-OE and *AtAGL16*-OE plants before and after drought stress treatment. Table S1: Primer sequences involved in this study.
