# Supplementary material for: GhAGL16 (AGAMOUS-LIKE16) Negatively Regulates Tolerance to Water Deficit in Transgenic Arabidopsis and Cotton
Source: Plants (Basel). 2024 Jan 18;13(2):282. doi: 10.3390/plants13020282 (PMC10820581; doi:10.3390/plants13020282)
Supplement: Supplementary file 1 [file plants-13-00282-s001.zip › plants-2784408-supplementary.pdf]

Supplementary material

GhAGL16 (*AGAMOUS-LIKE16*) Negatively Regulates Drought Tolerance in Transgenic *Arabidopsis* and Cotton

Jianfeng Lei [1], Yangzi You [2], Peihong Dai [2], Li Yu [2], Yue Li [2], Chao Liu [2] and Xiaodong Liu [2,*]

[1] College of Agronomy, Xinjiang Agricultural University, Nongda East Road, Urumqi 830052, China; kyleijianfeng@163.com

[2] College of Life Sciences, Xinjiang Agricultural University, Nongda East Road, Urumqi 830052, China; youyangzi@126.com (Y.Y.); peihong816@163.com (P.D.); yulixjnu@163.com (L.Y.); liyue6905@126.com (Y.L.); liuch_86@126.com (C.L.)

* Correspondence: xiaodongliu75@aliyun.com

The following Supplementary material is available for this article:

Figure S1. Phenotypes of *AtAGL16*-OE and WT plants before and after drought stress and after rehydration.

Figure S2. MDA, H_2O_2, and Pro contents as well as SOD and POD activities were determined in *GhAGL16*-OE and *AtAGL16*-OE plants before and after drought stress treatment.

Table S1. Primer sequences involved in this study

Figure S1. Phenotypes of *AtAGL16*-OE and WT plants before and after drought stress and after rehydration.

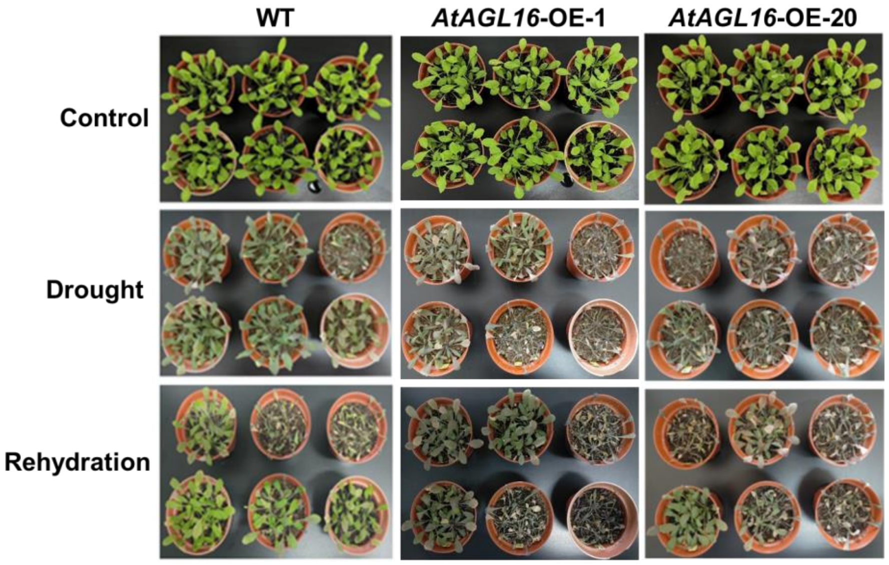

Figure S2. MDA, H_2O_2, and Pro contents as well as SOD and POD activities were determined in *GhAGL16*-OE and *AtAGL16*-OE plants before and after drought stress treatment.

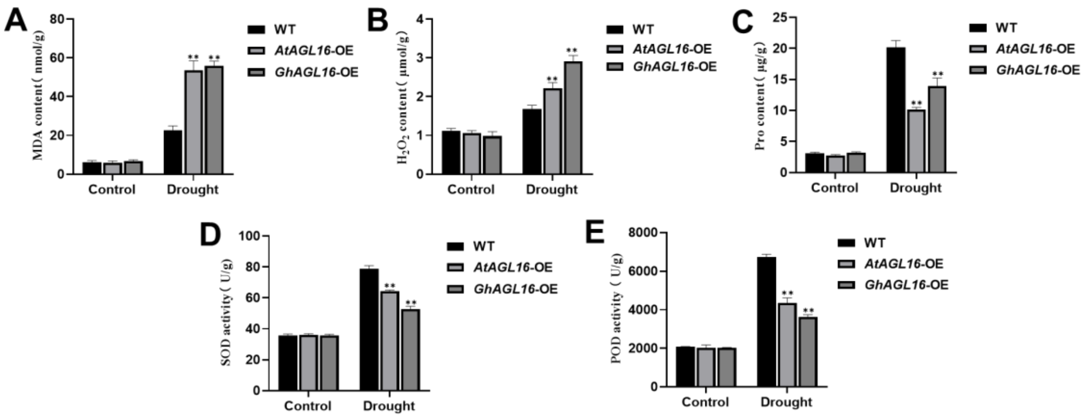

Values are the mean ± SD (n = 3 replicates). ** $p < 0.01$, (Student's t-test).

Table S1: Primer sequences involved in this study

Primer	Sequence(5'-3')	Application
Nco I-*GhAGL16*F:	CCATGGATGGGGAGAGGGAAAATAGT	Construction of *GhAGL16* subcellular localization vector
Bgl II-*GhAGL16*R:	AGATCTATGCAATTGCAATCCCAATTTTGTA	
*Eco*R I-*GhAGL16*F:	GAATTCATGGGGAGAGGGAAAATAGT	Construction of yeast vector
*Bam*H I-*GhAGL16*R:	GGATCCCTAATGCAATTGCAATCCCAATTTT	
Kpn I-*AtAGL16*F:	GGTACCATGGGAAGGGGCAAGATC	Construction of overexpression vector
*Bam*H I-*AtAGL16R:*	GGATCCTTATGCAATGAAGGAAAAATAGTTGAG	
Kpn I-*GhAGL16*F:	GGTACCATGGGGAGAGGGAAAATAGT	
*AtActin2*F:	GCACCCTGTTCTTCTTACCG	qPCR amplification of *Atactin2*

*AtActin2*R:	AACCCTCGTAGATTGGCACA	
Q-*GhAGL16*F:	GCGATGCTGAAGTTGGAGTA	qPCR analysis of *GhAGL16* mRNA accumulation
Q-*GhAGL16*R:	GGTTGTTGATGTTCCTCCTTTATTT	
Q-*AtAGL16*F:	ATGCTGAGGTTGGTGTCATC	qPCR analysis of *AtAGL16* mRNA accumulation
Q-*AtAGL16*R:	CCTTTGGCATCGCTGTATCT	
Xba I-*GhAGL16*F:	TCTAGATTGGAAATGAGCCTTCGTG	Construction of gene silencing vector
Kpn I-*GhAGL16*R:	GGTACCGTTTCATAGTTTTGCTGCTGTG	
*GhUBQ7*F:	GAAGGCATTCCACCTGACCAAC	qPCR amplification of *GhUBQ7*
*GhUBQ7*R:	CTTGACCTTCTTCTTCTTGTGCTTG	
Q-*GhCLA1*F:	GCCCTTTGTGCATCTTC	qPCR analysis of *GhCLA1* mRNA accumulation
Q-*GhCLA1*R:	CTCTAGGGGCATTGAAG	
*GhNCED1*F:	TCAGGATTCGCCAAAGTC	qPCR analysis of Marker gene mRNA accumulation
*GhNCED1*R:	AAGGCTCACCACCATACC	
*GhNCED3*F:	TGGGTTGAATCCCCTGATACTT	
*GhNCED3*R:	GCGACGTGTGGACTTACCTGT	
*GhNCED7*F:	TCCCAGTAGACGGTCCGACAAT	
*GhNCED7*R:	CAACTGGGGAACCACCGTGAAT	
*GhNCED9*F:	CCCAGTAGACGGTCCGATAA	
*GhNCED9*R:	ATCCCAAACCTTGACATCTTG	
*GhNCED14*F:	CTTCCCAAAACAGCTGACCCCA	
*GhNCED14*R:	AGAAATGATGTCCGGCGACAGG	
*GhZEP*F:	CTTCCCGCAAACAACATTCG	
*GhZEP*R:	GCCTTCCTCCTGCCATTATCA	
*GhABF4*F:	CCAGTCTTTCCAGAGCAGCA	
*GhABF4*R:	TCCCATCCCACCACTCTGAA	
*GhCYP707A*F:	TCTTTGAATCCCCCTTACACTTGA	
*GhCYP707A*R:	TGCCGCCATTGATGAACTGA	
